# Supplementary material for: The Combination of an mRNA Immunogen, a TLR7 Agonist and a PD1 Blocking Agent Enhances In-Vitro HIV T-Cell Immune Responses
Source: Vaccines (Basel). 2023 Jan 28;11(2):286. doi: 10.3390/vaccines11020286 (PMC9961394; doi:10.3390/vaccines11020286)
Supplement: Supplementary file 1 [file vaccines-11-00286-s001.zip › Supplementary Material.pdf]

Figure S1.

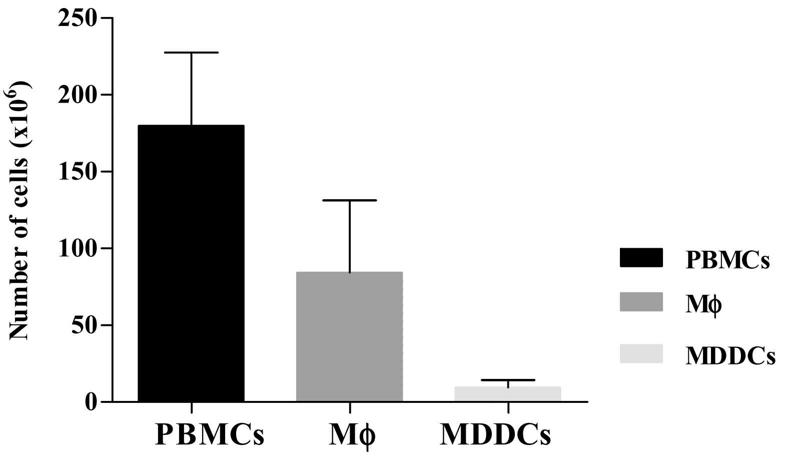

Figure S1. Efficiency of the monocytes yield and MDDC generation from the PBMCs of HIV infected patients. In general, the number of monocytes in PBMCs represented between 2-10% of the total number of PBMCs, although this can be very variable between patients. The graphic shows the percentage of monocytes (Mø) (dark grey bar) obtained after depletion of PBMCs from HIV-infected patients (Black bar) as well as the percentage of MDDCs generated (light grey bar) from the monocytes obtained (n=33).

Figure S2.

mAb's	Clone	
CD3	HIT3a	BD Pharmingen
CD4	SK3	BD Pharmingen
CD8	RPA-T8	BD Pharmingen
CD14	M5E2	BD Pharmingen
HLADR	G46-6	BD Pharmingen
CD86	2331	BD Pharmingen
CD80	L307	BD Pharmingen
CD83	HB15e	BD Pharmingen
CD40	5C3	Bionova
CCR7	2-L1-A	BD Pharmingen
PD1	EH12.1	BD Pharmingen

Figure S2. Antibodies used for flow cytometry. Informative table of clones and distributors of each monoclonal antibodies used for flow cytometry.

Figure S3.

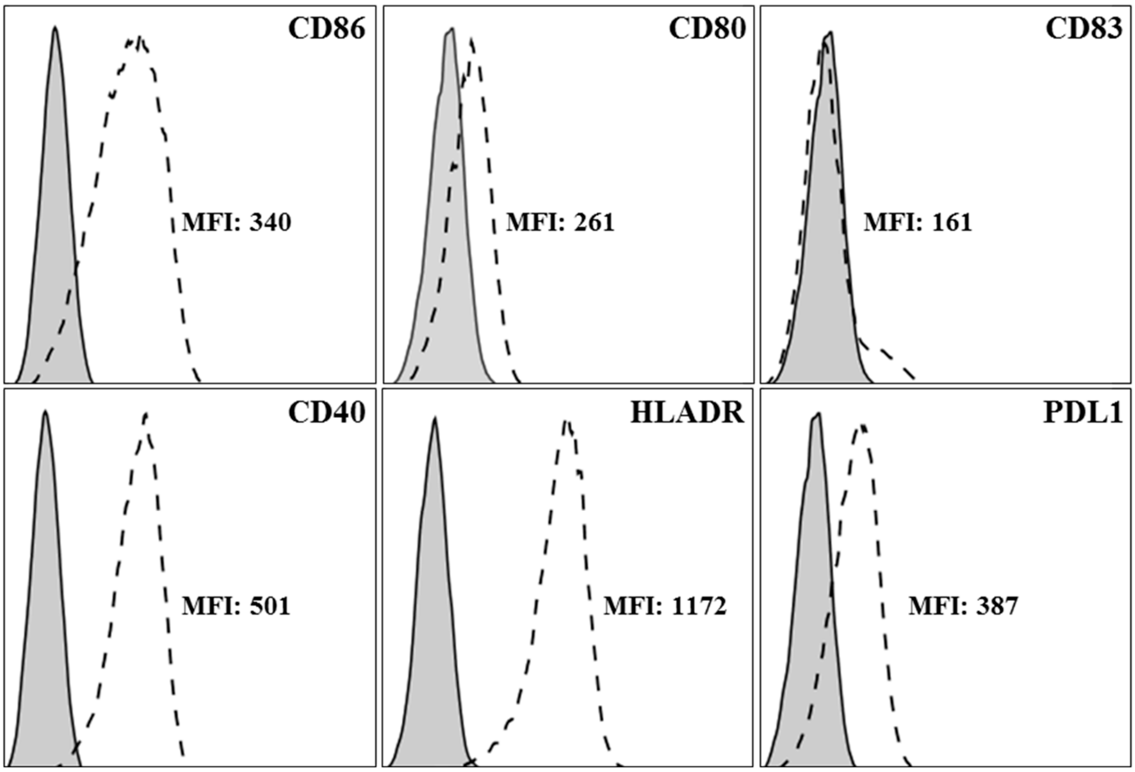

Figure S3. Phenotype of MDDCs obtained from HIV infected patients. Histograms showing the Mean Fluorescent Intensity (MFI) of an example of all the experiments performed of the phenotype of MDDCs from HIV infected patients gated for $CD14^-$ cells (n=17). Gray (Isotype, negative control), black dashed (marker).

Figure S4.

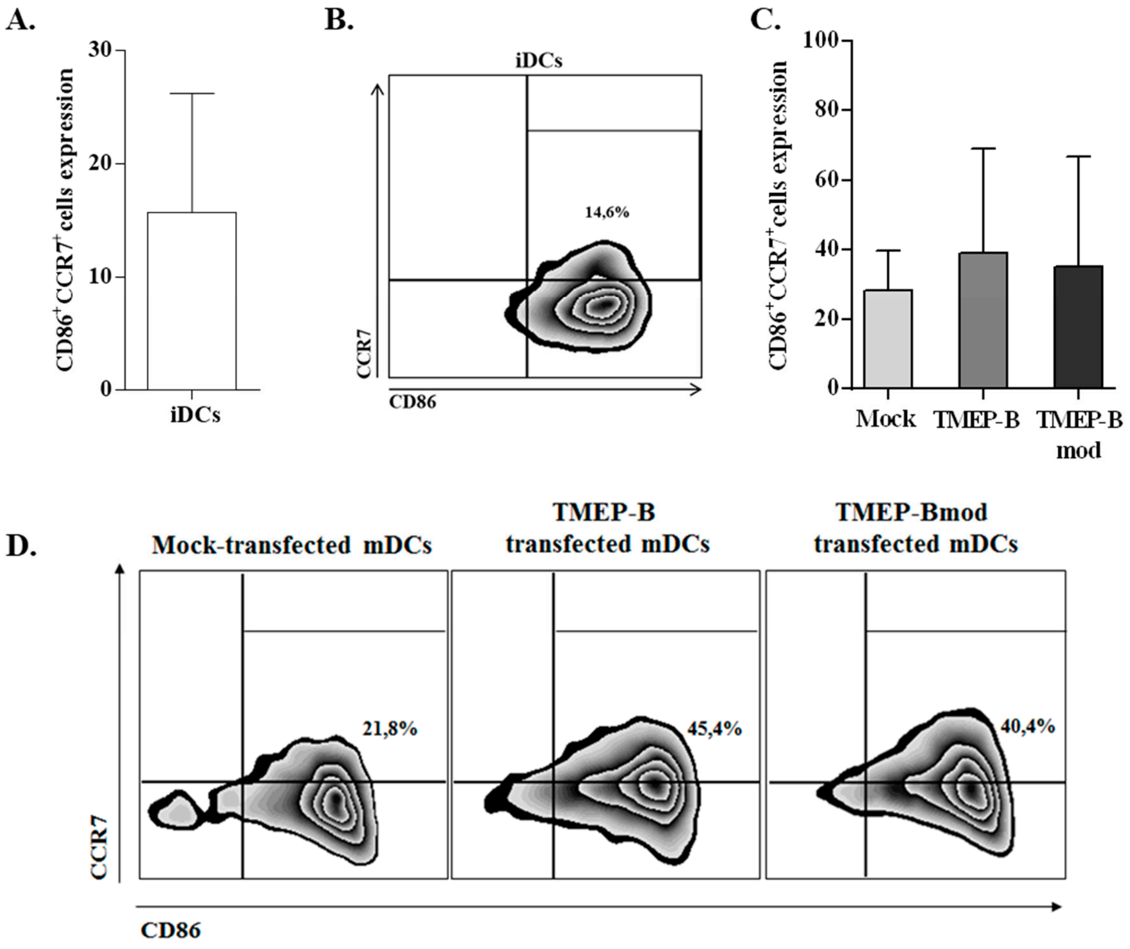

Figure S4. Analysis of CCR7 expression on DCs from HIV infected patients. Analysis of the $CD86^{+}CCR7^{+}$ expression on iDCs obtained from HIV infected patients (A-B), in Mock-transfected DCs (Negative control) and DCs electroporated with mRNA TMEP-B and/or mRNA TMEP-Bmod (C-D). The graphs A and C represent the mean of all the experiments performed (n=5). Graphic representation of the $CD86^{+}CCR7^{+}$ expression in one of the experiments performed, on MDDCs from HIV infected patients (B) and MDDCs electroporated with mRNA TMEP-B and TMEP-Bmod (D) (n=5). In the case of mDCs, the CCR7 expression was analyzed 24h after electroporation (Mock, TMEP-B and TMEP-Bmod). In all cases, cells were gated by $CD14^{-}$ and the cytokines maturation cocktail was added to help in the maturation of DCs. Statistical test used was Wilcoxon signed rank test (T-student, $p^{*}<0.05$; $p^{**}<0.01$; $p^{***}<0.001$).

Figure S5.

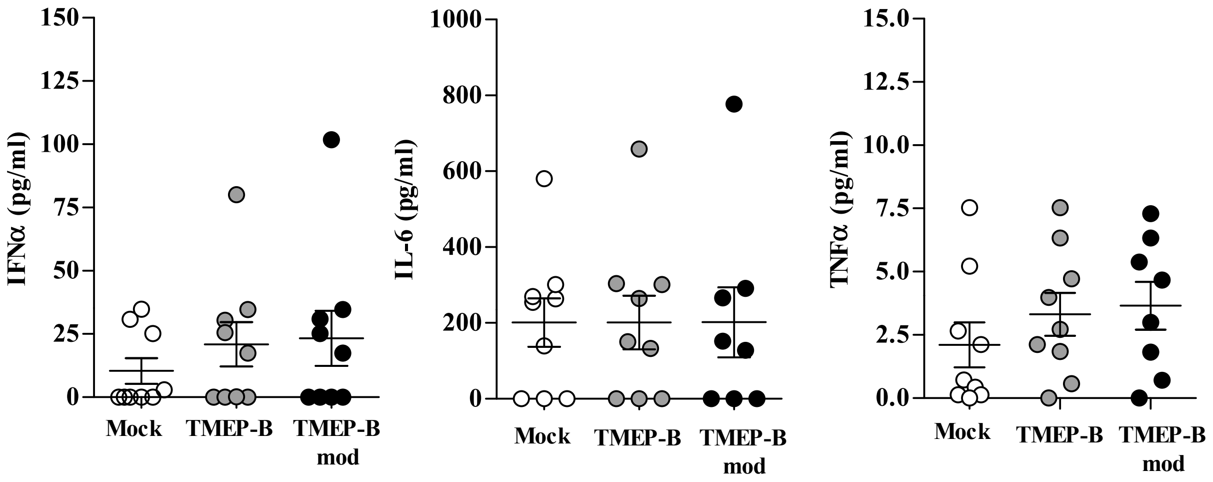

Figure S5. Study of cytokines secretion in the co-cultures of DC-T cell from HIV infected patients. Analysis of the cytokine secretion, IFNα, IL-6 and TNFα, after 6-days of co-cultures (DC: T cells) using DCs from HIV infected patients electroporated with TMEP-B (gray circle) or TMEP-Bmod (black circle) (n=9). Mock-transfected DCs were used as negative control (white circle). In all cases, the percentages ± SD are represented. Wilcoxon signed rank test was used for statistical analysis (*P<0.05; **P<0.01; ***P<0.001).

Figure S6.

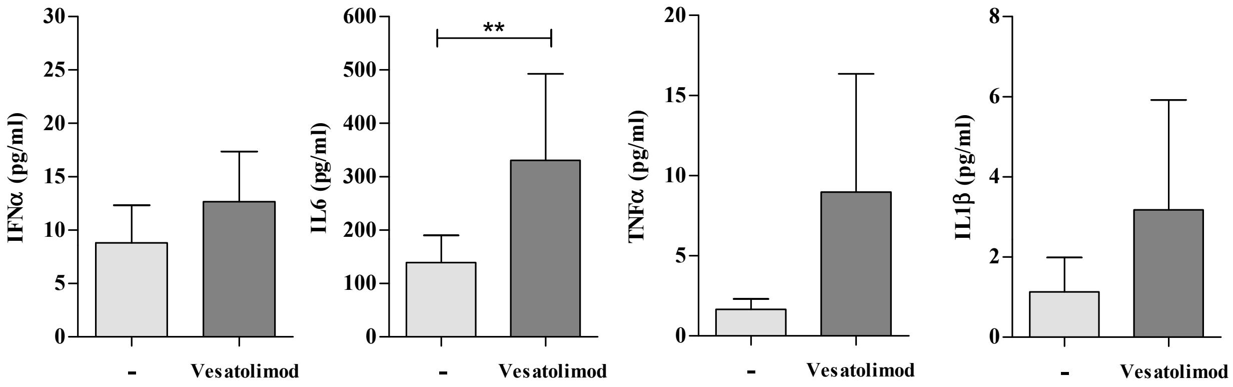

Figure S6. Study of cytokines secretion in absence or presence of Vesatolimod. Analysis of the cytokine secretion, IFNα, IL-6, TNFα and IL1β, after 6-days of co-cultures (DC: T cells) in absence or presence of Vesatolimod (1000nM) to confirm the activation of TLR7 pathway (n=13). In all cases, the percentages ± SD are represented. Wilcoxon signed rank test was used for statistical analysis (*P< 0.05; **P<0.01; ***P<0.001).
